# Methotrexate: should it still be considered for chronic calcium pyrophosphate crystal disease?

**DOI:** 10.1186/s13075-015-0598-1

**Published:** 2015-04-01

**Authors:** Eliseo Pascual, Mariano Andrés, Francisca Sivera

**Affiliations:** Sección de Reumatología, Hospital General Universitario de Alicante, Pintor Baeza 12, 03010 Alicante, Spain; Cátedra de Medicina (Reumatología), Universidad Miguel Hernández de Elche, Carretera Nacional 332, s/n. 03550 San Juan de Alicante, Alicante, Spain; Sección de Reumatología, Hospital General Universitario de Elda, Carretera Elda-Sax s/n, 03600 Elda, Spain

## Abstract

Chronic calcium pyrophosphate crystal arthritis is a clinical consequence of the formation and deposition of these crystals in joints and can result in persistent arthritis. Curative treatment would require the removal of crystals from joints and tissues, but to date all agents tested have proven ineffective. Management of the inflammatory manifestations of chronic calcium pyrophosphate disease includes glucocorticoids, non-steroidal anti-inflammatory drugs, or colchicine, and responses are usually satisfactory. However, in some patients, the response to these agents is poor or they are contraindicated. Methotrexate had been reported as a promising option in small case series; however, in a recent issue of *Arthritis Research & Therapy*, a clinical trial failed to confirm the anticipated benefits. Here, we discuss some issues that might have influenced the results of the study, before deciding to abandon methotrexate as a therapeutic option for patients with chronic calcium pyrophosphate arthritis.

## Editorial

We thank Finckh and colleagues [1] for their effort in performing a prospective controlled study of methotrexate (MTX) in patients with chronic calcium pyrophosphate (CPP) crystal arthritis, which is a less common clinical consequence of the formation and deposition of these crystals in joints and can result in persistent arthritis usually in older patients. Curative treatment of CPP disease (CPPD) would require the removal of crystals from joints and tissues, but to date all agents tested have proven ineffective [2]. Therefore, the management of CPPD relies on the control of the inflammatory manifestations, a scenario in which glucocorticoids, non-steroidal anti-inflammatory drugs, and colchicine can work well [3]. However, in some patients, the response to these agents is poor or contraindications for employing them arise, especially as CPPD occurs most often in older patients. Those with persistent disease despite traditional therapy constitute a troublesome subgroup of patients.

MTX seemed a promising option for these patients, as reported in small case series by Chollet-Janin and colleagues [4] and our group [5]. Conversely, other series from France noted no effects [6]. To clarify the effectiveness and safety of MTX, Finckh and colleagues [1] performed this clinical trial, which has failed to confirm the anticipated benefits. Before MTX is abandoned as a therapeutic option for patients with CPPD, it merits analyzing whether any methodological issue might have influenced the results of the trial.

In the study by Finckh and colleagues, they appear to have enrolled two quite different forms of CPPD: some with recurrent (more than three in a 6-month period) episodes of acute arthritis (likely separated by asymptomatic intercritical periods) and others with persistent, polyarthritis-like inflammatory disease. As the authors commented, the size of the study sample is lower than intended, as only 26 pairs were recruited (from the 28-pair calculated sample size) and only seven completed the study, performing an intention-to-treat analysis. Also, the authors selected DAS44 (44-joint disease activity score) as the main evaluation technique, and the sample size was also calculated on the basis of results of previous trials in rheumatoid arthritis (RA). However, around 25% of enrolled patients had recurrent CPP arthritis, and evaluations at the start of the study or both at the start and the end may have occurred during an intercritical asymptomatic period, hence the low DAS44 results and the lack of change. Only 12 patients showed a polyarticular presentation, the only group where these estimations would eventually apply. Therefore, the inappropriateness of the evaluation technique in a group and the low statistical power of the whole study must be taken into account when considering the results of the study.

The diagnosis of CPPD is performed on the basis of consistent clinical features and the identification of the crystals in synovial fluid (SF). In the case of episodes of acute arthritis, the relationship of the crystals with the joint inflammation appears clear, provided that no alternative diagnosis, such as an infection occurring at a joint containing CPP crystals, is reasonable. However, for polyarticular inflammatory disease, the relationship is less clear-cut. Chondrocalcinosis is a common finding in older age groups (approximately 18% in patients between 75 and 79 years old) [7], and the presence of crystals in SF of previously inflamed joints is constant [8]. In the absence of a negative association between CPP crystals and other diseases, we would expect that one in five patients who initiate a polyarthritis at this age would show chondrocalcinosis on X-rays and likely CPP crystals in their SF. Therefore, the presence of chondrocalcinosis or of a number of CPP crystals in SF in an older patient with persistent polyarthritis and features consistent with those of another disease—such as a seronegative RA (not a rare condition [9])—might imply the coexistence of the two disorders, RA being the main driver of inflammation; this could explain the possible response to MTX.

The study by Finckh and colleagues also outlines the difficulties of producing evidence in the treatment of less common diseases, especially when there are no economic interests in supporting the research. It also shows the difficulties posed when the clinical presentations are diverse and the reasons for considering them together or apart are not solidly grounded, thus hampering the interpretation of the results. Cross-over designs such as in this trial may facilitate valid conclusions in these circumstances.

We consider that this study should not be taken as definitive, and for those older patients with a seronegative symmetrical polyarthritis showing chondrocalcinosis or CPP crystals (or both) in their SF, MTX still merits a trial.

## Abbreviations

CPP: chronic calcium pyrophosphate
CPPD: chronic calcium pyrophosphate disease
DAS44: 44-joint disease activity score
MTX: methotrexate
RA: rheumatoid arthritis
SF: Synovial fluid
